# Navigating the Foggy Landscape of Epithelioid Peritoneal Mesothelioma: A Proposed Standardized Institutional Algorithm for Multimodal Management

**DOI:** 10.3390/cancers18132123

**Published:** 2026-06-30

**Authors:** Giorgio D’Annibale, Francesco Santullo, Lorenzo Barberis, Claudio Lodoli, Tommaso Partipilo, Giusi Giulia La Manna, Antonia Strippoli, Alexia Spring, Fabio Pacelli, Carlo Abatini

**Affiliations:** 1 Surgical Unit of Peritoneum and Retroperitoneum, Fondazione Policlinico Universitario A. Gemelli IRCCS, Largo Agostino Gemelli 8, 00168 Rome, Italy; giorgio.dannibale@guest.policlinicogemelli.it (G.D.); lorenzo.barberis@guest.policlinicogemelli.it (L.B.); claudio.lodoli@policlinicogemelli.it (C.L.); fabio.pacelli@policlinicogemelli.it (F.P.); carlo.abatini@policlinicogemelli.it (C.A.); 2 Catholic University of the Sacred Heart, General Surgery Residency Program, 00168 Rome, Italy; tommaso.partipilo@guest.policlinicogemelli.it (T.P.); giusigiulia.lamanna@guest.policlinicogemelli.it (G.G.L.M.); 3 Medical Oncology, Comprehensive Cancer Center, Fondazione Policlinico Universitario A. Gemelli IRCCS, Largo Agostino Gemelli 8, 00168 Rome, Italy; antonia.strippoli@policlinicogemelli.it (A.S.); aspring@scamilloforlanini.rm.it (A.S.)

**Keywords:** mesothelioma, peritoneum, HIPEC, cytoreductive surgery, PIPAC

## Abstract

**Purpose**: Diffuse malignant peritoneal mesothelioma is a rare disease requiring management in specialized referral centers. We report our institutional experience with epithelioid diffuse malignant peritoneal mesothelioma, focusing on a standardized treatment algorithm and on pre-operative cisplatin–pemetrexed as a bridge to cytoreductive surgery and HIPEC. **Methods**: Consecutive patients treated between 2016 and 2024 were retrospectively reviewed. Treatment allocation was based on diagnostic laparoscopy, PCI assessment, and multidisciplinary evaluation. Patients with technically resectable disease underwent upfront CRS–HIPEC, whereas borderline or unresectable patients received cisplatin–pemetrexed followed by systematic reassessment. **Results**: Forty-three patients were included; 19 underwent CRS–HIPEC, including 8 after pre-operative chemotherapy, and 24 received medical management alone. Curative-intent cytoreduction (CC-0/1) was achieved in all operated patients, with no 30-day mortality. No statistically significant difference in overall survival was detected between upfront and conversion surgery patients in this small cohort. Higher PCI and Ki-67 were independently associated with worse overall survival. **Conclusions**: A structured multidisciplinary pathway may support tailored treatment selection in epithelioid diffuse malignant peritoneal mesothelioma. In selected responders, cisplatin–pemetrexed may act as a bridge to surgery, although larger studies are required to validate this strategy.

## Simple Summary

Diffuse malignant peritoneal mesothelioma is a rare cancer that spreads along the lining of the abdominal cavity. Surgery combined with heated chemotherapy inside the abdomen can offer long-term disease control, but only in carefully selected patients. In this study, we describe the experience of a referral center using a structured pathway based on diagnostic laparoscopy, multidisciplinary evaluation, and tailored treatment selection. Patients suitable for complete tumor removal underwent surgery directly, whereas those initially considered not suitable received chemotherapy and were reassessed for possible surgery. Some patients responded sufficiently to become eligible for surgery, with outcomes comparable to those treated upfront. Our findings suggest that a standardized, surgery-centered strategy may help identify the best treatment for each patient and allow selected patients to benefit from delayed surgery after chemotherapy. Larger studies are needed to confirm these results.

## 1. Introduction

Diffuse malignant peritoneal mesothelioma (DMPM) is a rare malignancy arising from the mesothelial lining of the peritoneum, accounting for roughly 15–20% of all mesothelioma cases [1,2]. Unlike pleural mesothelioma, which is strongly associated with asbestos exposure and spreads primarily within the thoracic cavity, DMPM tends to remain confined to the abdomen with a low propensity for systemic metastatic spread [3,4,5].

However, DMPM encompasses a wide histopathological spectrum, and not all variants require or benefit from aggressive surgery. The epithelioid form, the most common variant, is generally amenable to cytoreductive surgery (CRS) and confers the best prognosis, whereas sarcomatoid and biphasic variants are associated with diffuse infiltration, chemoresistance, and poor outcomes [6].

Historically, epithelioid DMPM (eDMPM) carried a poor prognosis, with a median survival of 12–16 months under systemic therapy alone [3].

According to PSOGI/EURACAN and NCCN international guidelines [7,8], CRS + HIPEC remains the only potentially curative option in eDMPM, while systemic chemotherapy currently represents the most appropriate treatment in patients not eligible for radical surgery [9,10].

Moreover, growing evidence supports chemotherapy within a multimodal pathway as a bridge to surgery in selected patients. In those with high tumor burden or borderline resectability, pre-operative cisplatin–pemetrexed may achieve downstaging and provide a biological test of chemosensitivity, enabling conversion to curative-intent cytoreduction and improving prognosis [10,11,12]. However, the optimal sequencing of systemic therapy and surgery remains debated, as available evidence is largely retrospective and multicenter, with heterogeneous resectability criteria (PCI assessment method, visceral involvement, Ki-67 value, etc.) [13] and without a consistent definition of perioperative chemotherapy comparable to that applied in other solid tumors [14], limiting comparability across strategies and underscoring the need for clear, reproducible decision pathways. In this context, the role of pressurized intraperitoneal aerosol chemotherapy (PIPAC) remains controversial, and its position within the therapeutic algorithm may evolve with emerging studies and new clinical evidence [15,16].

This study describes the real-world experience of a national referral center in the multidisciplinary management of epithelioid diffuse malignant peritoneal mesothelioma, focusing on patient outcomes within a standardized institutional treatment algorithm and on the pragmatic integration of CRS-HIPEC, systemic chemotherapy, and PIPAC in routine clinical practice.

## 2. Materials and Methods

### 2.1. Study Design and Patient Selection

This was a retrospective, cohort study designed to describe an institutional diagnostic–therapeutic algorithm and to analyze short and long-term outcomes in patients affected by eDMPM treated between January 2016 and December 2024 at the Peritoneum and Retroperitoneum Surgical Unit of the Fondazione Policlinico Universitario A. At Gemelli IRCCS (Rome, Italy), the secondary aim was to evaluate the role of chemotherapy in patients not eligible for surgery as first-line treatment focusing on its function as “bridge to surgery” in selected cases.

Patients with sarcomatoid or biphasic histology, or those treated for recurrent disease after primary surgery were excluded. Demographic, clinical, surgical, and pathological data were extracted through an electronic health records review process, based on a standard workflow agreed in preliminary meetings among authors.

The study protocol was conducted in accordance with the ethical standards of the 1964 Declaration of Helsinki and was approved by the local Ethics Committee. All patients provided written informed consent for data collection and anonymous use for scientific purposes.

### 2.2. Diagnostic–Therapeutic Algorithm

**Figure 1:**
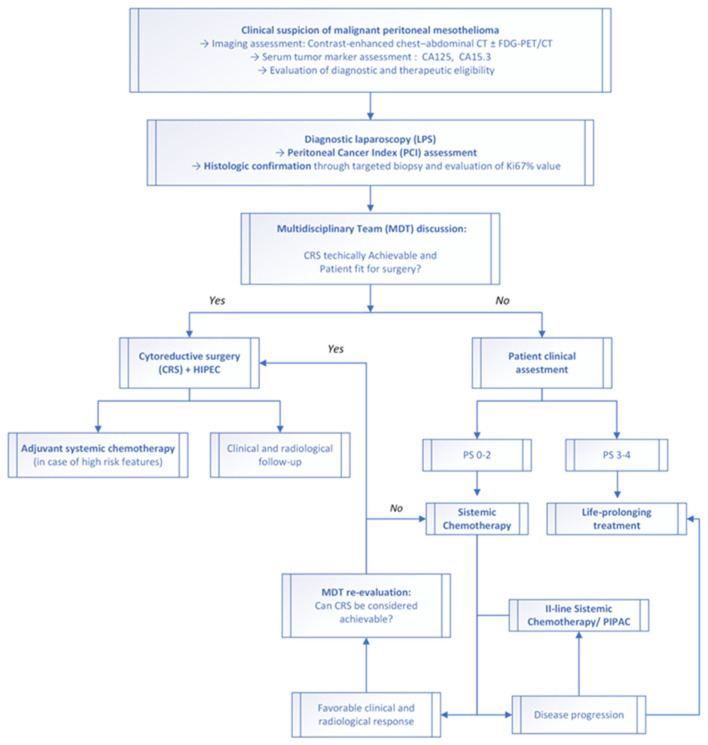
Diagnostic–therapeutic algorithm in management of primary epithelioid DMPM.

The diagnostic and therapeutic pathway adopted at our center in cases of suspected malignant peritoneal mesothelioma is illustrated in Figure 1.

The initial diagnostic work-up includes comprehensive clinical examination, standard laboratory tests, and assessment of serum tumor markers (CA 125, CA 15-3). A contrast-enhanced CT scan of the chest, abdomen, and pelvis is performed in all cases to define the extent of disease, while FDG-PET/CT is reserved for selected cases in which extra-abdominal spread or ambiguous findings are suspected.

Based on this preliminary assessment, diagnostic laparoscopy (LPS) is performed to obtain targeted biopsies for expert histological confirmation and to assess peritoneal disease burden by calculating the Peritoneal Cancer Index (PCI) (24).

After eDMPM is confirmed, these findings are subsequently reviewed by a multidisciplinary team (MDT) comprising peritoneal surgeons, medical oncologists, radiologists, and pathologists experienced in peritoneal surface malignancies.

Two different scenarios can therefore be drawn:

(1) If curative-intent cytoreductive surgery (CRS) is considered technically achievable and the patient is fit for surgery, treatment proceeds with CRS combined with hyperthermic intraperitoneal chemotherapy (HIPEC).
(2) When CRS is judged not technically feasible, the therapeutic decision is guided by the patient’s performance status (PS) [17].
  a. Patients with acceptable PS (0–2) receive first-line cisplatin–pemetrexed to pursue downstaging, followed by re-evaluation after 3–4 cycles; responders with technically feasible cytoreduction proceed to CRS ± HIPEC with curative intent, whereas progressive disease is managed with second-line and/or palliative systemic therapy, with PIPAC considered as a palliative option.
  b. Patients with poor performance status (PS ≥ 3–4), who are unsuitable for either surgery or systemic therapy, are managed with life-prolonging treatments focused on symptom relief and quality of life.

Disease is considered not technically suitable for upfront CRS when complete or near-complete cytoreduction with curative intent (CC-0/1) is judged unlikely based on radiological or laparoscopic assessment. The main criteria include extensive small-bowel serosal or mesenteric involvement, diffuse involvement of the mesenteric root, deep plaques involving the porta hepatis or hepatoduodenal ligament, or a disease distribution requiring visceral resections considered incompatible with a safe surgical procedure.

Radiological evidence of extra-abdominal disease, nodal metastases, and large bulky retroperitoneal masses are considered exclusion criteria for surgical indication. In case of radiological suspicion of nodal/retroperitoneal disease, a CT-guided fine needle biopsy is performed whenever feasible. Adjuvant systemic chemotherapy is performed in the presence of high-risk pathological or biological features (PCI > 17 or Ki-67 > 9%).

All patients are subsequently enrolled in a structured follow-up program at our institution, consisting of outpatient visits every three to four months during the first two years and every six months thereafter. Each follow-up includes clinical assessment, tumor-marker testing, and contrast-enhanced CT imaging to detect recurrence or progression.

### 2.3. Surgical Techniques

#### 2.3.1. CRS + HIPEC

Surgical cytoreduction was performed according to Sugarbaker’s principles of peritonectomy [18]. The extent of resection was tailored to the intra-abdominal disease distribution observed at laparotomy. Procedures included total parietal peritonectomy, omentectomy, diaphragmatic peritonectomy, pelvic peritonectomy, and visceral resections when necessary to achieve macroscopic tumor clearance. Gynecological procedures (hysterectomy and oophorectomy) were routinely performed, except in young women wishing to preserve fertility, following dedicated counselling. Protective stomas (ileostomy or colostomy) were created at the discretion of the operating surgeon, according to intraoperative findings and the anticipated risk of anastomotic complications.

All procedures were performed or supervised by a surgeon who completed a structured learning-curve program to ensure adequate surgical proficiency in CRS and HIPEC procedures [19].

According with international guidelines, completeness of cytoreduction (CC) 0–1 was considered the goal to define a curative surgical treatment [7,20].

Following CRS, all eligible patients received HIPEC using a closed-abdomen technique with a four-catheter perfusion circuit. As defined by PSOGI Consensus guidelines [21], the perfusate consisted of cisplatin 50 mg/m^2^ and doxorubicin 15 mg/m^2^. Perfusion was maintained at a target intraperitoneal temperature of 42 °C for 60 min, and nephroprotection was achieved through perioperative intravenous hydration (initiated 12 h before and continued after HIPEC) and sodium thiosulfate administration (9 g/m^2^ IV bolus at time 0, followed by a continuous infusion of 1.2 g/m^2^/h for 6 h).

#### 2.3.2. Pressurized Intraperitoneal Aerosolized Chemotherapy (PIPAC)

PIPAC was conducted laparoscopically under general anesthesia. A nebulizer (Capnopen-MIP, Reger Medizintechnik, Rottweil, Germany) connected to a high-pressure injector was employed to deliver a pressurized aerosol of cytotoxic agents into the peritoneal cavity. The procedure was visualized through a laparoscopic camera fixed with a self-retaining retractor, while the drug administration was remotely controlled to minimize occupational exposure. The pneumoperitoneum was maintained for 30 min at 37 °C, after which the aerosol was evacuated through a closed waste system. Initially, PIPAC was carried out using cisplatin at 7.5 mg/m^2^ and doxorubicin at 1.5 mg/m^2^. Following the publication of the phase I study by Tempfer et al. in 2018 [16], the protocol was updated to the recommended doses of 10.5 mg/m^2^ for cisplatin and 2.1 mg/m^2^ for doxorubicin, which were subsequently adopted in our practice.

### 2.4. Data Collection

Pre-operative variables included age, sex, performance status (ECOG), ASA score, body mass index (BMI). Disease-related variables included PCI, histological subtype, proliferative index (Ki-67%), and the administration of pre-operative or adjuvant chemotherapy. Operative details comprised the number and type of peritonectomy procedures, visceral resections performed, operative time, estimated blood loss, completeness of cytoreduction (CC score). Post-operative complications were divided into minor (Clavien–Dindo I–II), and major (Clavien–Dindo ≥ III) [22,23]. Length of hospital stay and 30-day mortality were recorded as post-operative variables.

For the purposes of outcome analysis, patients were stratified into two main treatment cohorts according to the therapeutic strategy

1. Cytoreductive surgery group (CRS-G): including all patients who ultimately underwent CRS ± HIPEC. Based on the initial treatment pathway, this cohort was further subdivided into two subgroups:
  (i) Upfront surgery group (uS-G): comprising patients treated with primary CRS ± HIPEC as first-line therapy according to MDT indication.
  (ii) Conversion surgery group (cS-G): including patients initially deemed unresectable who received systemic chemotherapy and/or PIPAC, underwent structured re-evaluation (imaging and MDT review, with repeat laparoscopy when indicated), and subsequently proceeded to CRS ± HIPEC.

2. Medical-management group (MM-G): including patients who never underwent CRS and were managed with systemic chemotherapy, PIPAC, or life-prolonging treatment with palliative intent.

Overall survival (OS) was defined as the interval between diagnosis and the date of death from any cause or censored at the last follow-up. Disease-free survival (DFS) was calculated only for patients undergoing CRS–HIPEC and defined as the time from CRS–HIPEC to radiological or histological recurrence, or last follow-up. For patients managed without CRS–HIPEC, progression-free survival (PFS) was recorded descriptively as the time from initiation of systemic therapy to radiological progression and was not included in inferential survival analyses.

### 2.5. Statistical Analysis

Continuous variables were expressed as median values with interquartile ranges (IQRs) or mean ± standard deviation, depending on distribution, while categorical variables were expressed as frequencies and percentages.

Statistical analysis was primarily focused on the CRS–HIPEC cohort. The Cox proportional hazards model was used for univariate analysis to explore potential prognostic factors associated with survival outcomes. Variables demonstrating a *p*-value of <0.10 at univariate testing were subsequently entered into a multivariate model. A backward stepwise elimination procedure based on likelihood ratio statistics was then applied to identify independent prognostic determinants, with statistical significance defined as *p* ≤ 0.05. Because of the small number of events, multivariable analyses should be interpreted as exploratory.

Survival curves for OS and DFS/PFS were estimated using the Kaplan–Meier method. Survival analyses were conducted between groups of patients with different treatment and Kaplan–Meier curves were generated to illustrate survival distributions within each subgroup. Differences between curves were assessed using the log-rank test. Statistical significance was defined with *p* < 0.05.

All analyses were performed using IBM SPSS Statistics version 29.0 (IBM Corp., Armonk, NY, USA).

## 3. Results

### 3.1. Patient Characteristics

A total amount of 43 patients with histologically confirmed eDMPM were included and stratified into two main cohorts according to the initial management strategy: CRS-G (*n* = 19, 44.2%) and MM-G (*n* = 24, 55.8%).

Within the CRS-G, 11 patients (57.9%) were recorded into the uS-G, while 8 patients (42.1%) received pre-operative cisplatin–pemetrexed chemotherapy as firstly judged not eligible for upfront CRS. Those were enrolled in the cS-G after disease downstaging allowing surgical treatment. Baseline demographic, clinical, and pathological data for both groups are detailed in Table 1. The median follow-up of the whole cohort was 48 months.

### 3.2. CRS-G: Intra and Post-Operative Details

All 19 patients in the CRS-G underwent CRS + HIPEC. The mean operative time was 603 min (389–911). Among the 19 patients treated with CRS–HIPEC, complete cytoreduction (CC-0) was achieved in 11 patients (57.9%), while near-complete cytoreduction (CC-1) was achieved in 8 patients (42.1%). Overall, curative-intent cytoreduction (CC-0/1) was achieved in all 19 patients (Table 2).

Intraoperative complications were reported in 8 patients (42.1%), mostly minor injuries such as diaphragmatic (21%), or bladder laceration (21%), all repaired during the same procedure without sequelae. Diaphragmatic lacerations were repaired before HIPEC with direct suturing after an air leak bubble test. Bladder injuries were repaired intra-operatively with a double-layer absorbable suture. In one patient (5.3%), a right hepatic artery injury was identified intraoperatively and repaired with Prolene sutures. In one case (5.3%), a vaginal laceration was detected and repaired using a continuous absorbable suture. Post-operative complications occurred in 11 patients (57.9%). Major morbidity (Clavien–Dindo ≥ III) was observed in 5 patients (26.3%), while the remaining 6 (31.7%) experienced minor events managed conservatively. Details of the post-operative complications are described in Table 3. No 30-day mortality was recorded.

### 3.3. CRS-G: Survival Outcomes and Predictive Factors

**Figure 2:**
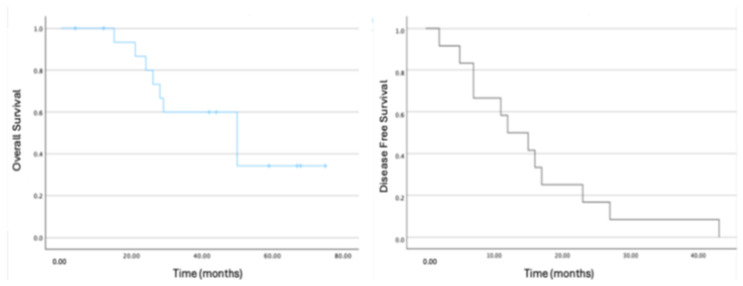
Overall survival and disease-free survival in patients treated with CRS (KM curves).

For patients who underwent CRS + HIPEC, the median overall survival (OS) was 50 months (ranging from 4 to 75), while the median DFS was 12 (ranging from 2 to 43) (Figure 2).

**Figure 3:**
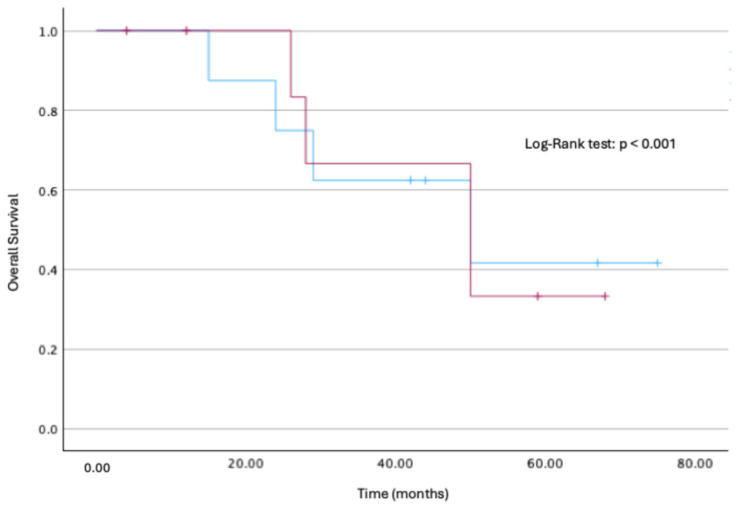
Kaplan–Meier OS curves according to the initial treatment pathway. uS-G (red line). cS-G (blue line). Log-rank *p* = 0.125.

Within the CRS-G, a survival analysis was performed to better delineate prognostic differences between uS-G and cS-G; The Kaplan–Meier analysis showed that no statistically significant difference in OS was detected between the upfront surgery group and the conversion surgery group in this small cohort (median OS = 50 months in both groups; log-rank *p* = 0.125) (Figure 3).

As shown in Table 4, univariate Cox regression analysis identified PCI and Ki-67 proliferative index as variables associated with OS. Both PCI and Ki-67 retained statistical significance in the multivariate model [PCI: HR 1.195, 95% CI: 1.038–1.375, *p* = 0.013; Ki-67: HR 1.169, 95% CI: 1.030–1.382, *p* = 0.019]. No statistically significant associations with survival were found for administration of pre-operative chemotherapy or intra/post-operative complications.

## 4. Discussion

This study provides a real-world snapshot of how patients with eDMPM can be managed longitudinally within a dedicated referral center, through a standardized multidisciplinary pathway integrating cytoreductive surgery, systemic chemotherapy, and PIPAC.

As the largest multi-institutional series available, Kepenekian’s RENAPE multicenter experience has provided invaluable insights into the role of different treatments in case of eDMPM [13]. Specifically, pre-operative chemotherapy was associated with inferior outcomes and emerged as an independent negative prognostic factor. However, several methodological aspects warrant consideration: baseline disease burden may have been less precisely assessed when staging laparoscopy was not routinely performed; indications for pre-operative chemotherapy differed across centers (e.g., borderline resectability, referral delays, other reasons), generating a heterogeneous cohort; and patients with good responses who later received post-operative chemotherapy were reclassified into the “perioperative chemotherapy” group, potentially leaving the pre-operative cohort enriched with poorer-prognosis cases while shifting favorable responders elsewhere. Together, these sources of selection and classification bias likely contributed to the inferior survival observed in the pre-operative arm.

The propensity-matched analysis by Chatterjee et al. [24] further supports principles central to our institutional strategy. Complete CRS–HIPEC remains the cornerstone of curative treatment in eDMPM. Their data also favor adjuvant chemotherapy in the presence of high-risk features, while questioning routine neoadjuvant therapy outside clearly unfavorable scenarios. Although our cohort is smaller and limited to epithelioid histology, both series convey a consistent message: best outcomes are achieved when perioperative systemic therapy is embedded within a pathway centered on curative intent cytoreduction, careful pre-treatment stratification, and early referral to specialized peritoneal surface malignancy units.

In our institutional model (Figure 1), therapeutic allocation is driven by a feasibility-based approach centered on laparoscopic assessment: patients with cytoreducible disease (anticipated CC-0/1) proceed to upfront CRS–HIPEC, whereas those with non-cytoreducible disease receive first-line cisplatin–pemetrexed and undergo structured reassessment (imaging, MDT review, and repeat laparoscopy when indicated). Only patients in whom downsizing renders curative-intent cytoreduction technically achievable are offered delayed CRS–HIPEC aligning our strategy with a “conversion therapy” concept [11].

One of the most relevant findings of this study is that 25% (8/32) of patients who received chemotherapy experienced sufficient disease regression to be converted to a technically resectable status and subsequently underwent CRS–HIPEC. Overall survival did not differ significantly between the upfront-surgery group (uS-G) and the conversion-surgery group (cS-G). In addition, adjuvant chemotherapy was systematically offered to all patients undergoing CRS–HIPEC, ensuring broadly comparable post-operative systemic treatment across groups. Collectively, these data indicate that, when pre-operative systemic therapy is applied selectively and followed by rigorous radiologic and multidisciplinary reassessment before proceeding to CRS–HIPEC, a conversion strategy may achieve long-term outcomes that are not demonstrably inferior to those observed with an upfront-surgery approach, although residual confounding and selection bias cannot be excluded. Given the small sample size of the upfront surgery and conversion surgery subgroups, this finding should be interpreted with caution. Therefore, these results should be considered exploratory and require validation in larger multicenter cohorts.

Considering post-operative outcomes, the low morbidity and absence of mortality in this cohort are in line with published reports and further support the value of treating these complex cases in high-volume referral centers [3,25,26,27,28,29].

Despite their negative prognostic implications [30,31], our hypothesis is that high PCI or elevated Ki-67 should not be considered absolute contraindications to CRS–HIPEC in anatomically resectable patients, as guidelines consider these factors relative rather than exclusionary when complete cytoreduction is achievable [32]. This is particularly relevant given the modest activity of systemic chemotherapy (response rates: 15–40%) [33,34] and the evidence that prolonged survival is still possible in “high-risk” patients after complete cytoreduction is achieved [27,29,35,36].

In this context, although our cohort size is limited, the standardized therapeutic pathway implemented at our institution offers a coherent framework that may enhance the interpretability of our findings. This structured approach is intended to reduce treatment heterogeneity, support timely surgical decision-making and favor selection of patients with at least objective disease control for consideration of surgery. Within this setting, pre-operative therapy can be viewed as a complementary component of care rather than an alternative competing strategy, and it may partly explain why we did not observe an apparent adverse impact on long-term outcomes in our cohort.

It is important to acknowledge the limitations of our study. The CRS-G and MM-G were not intended to be compared as equivalent treatment cohorts, and data from the MM-G were reported for descriptive purposes only. Indeed, the MM-G included older and clinically more fragile patients, often unsuitable for aggressive surgery because of poor performance status, frailty, or unresectable disease. Therefore, the shorter survival observed in this group should not be interpreted as the isolated effect of medical treatment alone, but rather as the result of treatment allocation and baseline patient- and disease-related factors. Moreover, our analysis is retrospective and based on a single-center experience. Reflecting the rarity of DMPM, our cohort has a relatively limited sample size, which may constrain the statistical power and generalizability of our findings. Accordingly, survival analyses should be interpreted with caution. Furthermore, there is a potential selection bias inherent to referral-center cohorts, as our institution may have attracted patients with better performance status or greater suitability for aggressive multimodality therapy. In this context, larger and preferably multicenter studies will be needed to validate our findings and further refine treatment strategies for this rare disease.

## 5. Conclusions

Within a highly standardized institutional pathway, CRS–HIPEC for epithelioid DMPM achieved favorable long-term survival, even in patients initially deemed unresectable but converted to surgery after cisplatin–pemetrexed. These findings support a surgery-centered strategy in which chemotherapy may act as a complementary tool for conversion to resectability in a selected subset of patients. Larger multicenter studies are needed to validate this algorithm and refine patients’ selection criteria in this rare disease.

## Figures and Tables

**Table 1:** Patient demographic and pathological characteristics. CRS-G on the left; MM-G on the right.

Variable	CRS-G 19 Patients *N* (%)	MM-G 24 Patients *N* (%)
**Age, median (range)**	59 (11–77)	73 (38–91)
**Gender, *n* (%)**		
Male	5 (26.3)	10 (41.7)
Female	14 (73.7)	14 (58.3)
**BMI**		
<18	1 (5.3)	1 (4.2)
18–30	18 (94.7)	21 (87.5)
>30	0 (0.0)	2 (8.3)
**ASA, *n* (%)**		
Score < 3	19 (100)	22 (91.7)
Score ≥ 3	0 (0)	2 (8.3)
**ECOG, PS *n* (%)**		
0–2	19 (100)	19 (79.2)
3–4	0 (0)	5 (20.8)
**Staging laparoscopy, *n* (%)**		
Yes	19 (100)	24 (100)
No	0 (0)	0
**PCI median (range)**	19 (5–35)	22 (8–39)
**Ki67**		
<9%	5 (26.3)	5 (20.8)
>9%	14 (73.7)	19 (79.2)
**Median OS (min** **–** **max)**	50 (4–75)	7 (1–49)
**Median DFS (CRS-G)/PFS (MM-G)**	12 (2–43)	5 (2–29)

**Table 2:** Operative details in the surgical cohort (CRS-G).

Variable	19 Patients *N* (%)
**Timing of surgery**	
Upfront surgery group (uS-G)	11 (57.9)
Conversion surgery group (cS-G)	8 (42.1)
**Completeness of cytoreduction, *n* (%)**	
Completed with curative intent (CC: 0–1)	19 (100)
Incomplete cytoreduction (CC: 2–3)	0 (0)
**Adjuvant chemotherapy**	19 (100)
**Operative time median (range)**	603 (389–911)
**Selective mesenterectomy, *n* (%)**	
Yes	12 (63.2)
No	7 (36.8)
**Omentectomy, *n* (%)**	
Yes	19 (100)
No	0 (0)
**Gastric resection, *n* (%)**	
Yes	2 (10.5)
No	17 (89.5)
**Splenectomy, *n* (%)**	
Yes	3 (15.8)
No	16 (84.2)
**Small bowel resection, *n* (%)**	
Yes	3 (15.8)
No	16 (84.2)
**Colorectal resection, *n* (%)**	
Right hemicolectomy	5 (26.3)
Left hemicolectomy/RAR	13 (68.4)
**Hysterectomy/oophorectomy, *n* (%)**	
Yes	10 (52.6)
No	9 (47.4)
**Loop ileostomy, *n* (%)**	
Yes	10 (52.6)
No	9 (47.4)
**Completeness of cytoreduction, *n* (%)**	
Complete (CC: 0–1)	19 (100)
Uncomplete (CC: 2–3)	0 (0)
**HIPEC**	
Yes	19 (100)
No	0 (0)

**Table 3:** Morbidity and mortality in CRS-G.

Variable	19 Patients *N* (%)
**Post-operative complication, *n* (%)**	
Yes	11 (57.9)
No	9 (47.4)
**Clavien–Dindo grade, *n* (%)**	
I	3 (15.8)
II	3 (15.8)
IIIa	3 (15.8)
IIIb	1 (5.3)
IV	1 (5.3)
V	0 (0)
**Major complications (CD ≥ III)**	5 (26.3)
**Type of post-operative complication, *n* (%)**	
Pulmonary complications (pleural effusion/pneumonia)	5 (26.3)
Wound infection	3 (15.8)
Intra-abdominal bleeding	2 (10.5)
Abdominal collection	2 (10.5)
Post-operative ileus	1 (5.3)
Urinary tract infection	1 (5.3)
Anastomotic leak	1 (5.3)
Acute kidney injury (AKI)	1 (5.3)
**30-days mortality**	0 (0)

**Table 4:** Predictive factors for OS in CRS-G, univariate and multivariate survival analysis.

Variables	HR	Univariate Analysis	*p*-Value	HR	Multivariate Analysis	*p*-Value
		95% CI			95% CI	
Pre-operative chemotherapy	1.021	[0.252–4.131]	0.977			
PCI score	1.165	[1.029–1.320]	0.016	1.195	[1.038–1.375]	0.013
Intra-operative complications	0.959	[0.232–3.965]	0.954			
Major post-operative complications	0.220	[0.025–6.662]	0.529			
Ki-67%	1.141	[1.001–1.301]	0.047	1.169	[1.030–1.382]	0.019

## Data Availability

The data presented in this study are available on reasonable request from the corresponding author, subject to institutional approval. The data are not publicly available due to privacy and ethical restrictions related to the retrospective use of anonymized clinical patient data and institutional regulations on patient confidentiality.
